# Formation of Corrugated Damage on Bearing Race under Different AC Shaft Voltages

**DOI:** 10.3390/ma17040859

**Published:** 2024-02-12

**Authors:** Zhihao Lou, Chenfei Song, Yulong Ren, Xianjuan Pang, Huanhuan Lu, Sanming Du, Yongzhen Zhang

**Affiliations:** National United Engineering Laboratory for Advanced Bearing Tribology, Henan University of Science and Technology, Luoyang 471023, China; 210321020249@stu.haust.edu.cn (Z.L.); 220320020238@stu.haust.edu.cn (Y.R.); xjpang2001@haust.edu.cn (X.P.); luhuanuan@haust.edu.cn (H.L.); dsming@haust.edu.cn (S.D.); yzzhang@haust.edu.cn (Y.Z.)

**Keywords:** corrugated damage, equivalent resistance, electrical erosion, lubrication leakage, hardness

## Abstract

Corrugated damage to bearings is a common fault in electrical facilities such as new energy vehicles, wind power, and high-speed railways. The aim of this article is to reveal the microscopic characteristics and formation mechanism of such damages. The corrugation with alternating “light” and “dark” shape was produced on GCr15 bearing races in the experimental conditions. Compared to the light area, the dark area (in the images generated by optical microscope) has more severe electrical erosion, lower hardness, more concave morphology, and lower oxidation. As the voltage increases, the width of the corrugation, the height difference between corrugation, and surface roughness all increase. It is believed that the formation of corrugated damage requires a sufficiently high voltage to induce the periodic destruction and reconstruction of the lubrication film. When the bearing is in a metal-lubrication film–metal contact state, the high voltage causes the lubrication film to break down and induce electrical erosion. Then, the contact area is in metal–metal contact, and the surface is mainly damaged by mechanical rolling. After the reconstruction of lubrication film, the next round of electrical erosion begins. The results are helpful for a deeper understanding of the mechanism of bearing erosion in electrical application.

## 1. Introduction

Bearings are fundamental mechanical components that perform functions such as loading, rotation, and friction reduction. With the rapid development of new energy vehicles, wind power systems, high-speed railways, and other electromechanical drive equipment, motor bearings are forced to operate in complex electromagnetic fields. Many researchers have observed that an electric field between the inner and outer rings can lead to electrical erosion of the bearing surfaces [1]. Electrical damage to bearings has become a critical problem that affects the safe operation of electric motor systems [2,3,4]. Therefore, the mechanism of the electrical erosion of bearings has been extensively studied.

Bearing electrical erosion occurs in various forms, such as morphological damage, lubrication failure, and material degradation [5]. Corrugated damage is a distinctive erosion morphology on bearing surfaces. Liu et al. [6] observed that the corrugated damage consisted of a series of scratches of different densities, with dense scratches constituting “dark stripes” and sparser scratches constituting “bright stripes”. Zika et al. [7] believed that low-frequency bearing currents caused damage to the bearing raceways and rolling elements, eventually leading to macroscopic bright and dark rubbing-board-like ripples. Feng et al. [4] suggested that grooves are formed under the action of periodic currents and appear as bright and dark-spaced streaks under light microscopy. Ma et al. [8] concluded that the structure of the dark area of corrugated damage was relatively dense with low roughness, the microstructure of the light area was sparse with high roughness, and the thickness of the melt layer in the light area was thicker than that in the dark area. Oliver et al. [9] concluded that corrugated damage was caused by the mechanical resonance of the rolling element passing through the erosion pits. Karolina et al. [10] believed that shaft currents which were caused by excessive load usually occurred in the weak lubrication area, where the grooves were manifested as multiple lines. The above studies focused on the morphological characteristics of corrugated damage with alternating forms of light and dark. Owing to different imaging mechanisms, the same area may exhibit inconsistent contrast under different microscopes, resulting in a lack of comparability between the research results of different studies. Furthermore, the microscopic non-uniform characteristics such as morphology, composition, and mechanical properties of the “light” and “dark” areas have not been distinguished.

In the literature [11,12], we have studied the electric erosion on lubricated rolling surface under DC and 50 Hz AC conditions. The corrugated damage cannot be induced by changing the bearing speed and shaft voltage, and only random erosion pits are observed. However, these results have shown that bearing erosion under AC conditions was more pronounced than that under DC conditions. It is speculated that the skin-effect at high frequencies could cause charges to accumulate on the surface, making the lubricating film more susceptible to breakdown. After continuous attempts, corrugated damage could be reliably reproduced under our experimental conditions when the AC frequency was increased to 400 Hz. In our previous study of dry sliding with current or dry rolling with current, erosion was often observed but corrugated damage has never been observed [11,12,13]. Therefore, corrugated damage should be a unique damage mode under lubricated current-carrying rolling conditions.

Corrugated damage to bearings has attracted attention in engineering applications such as new energy vehicles and wind power. Researchers explore the reasons from multiple perspectives such as bearing structure, frequency conversion control, and electromagnetic induction. Bearings are typical friction pairs that transform into current-carrying friction pairs under lubrication conditions after conduction. The present research on current-carrying tribology mainly focuses on dry conditions such as pantographs and brushes [14,15]. This article conducts research on current-carrying tribology under lubrication conditions, which may be a beneficial supplement to the theory of tribology. From the perspective of tribology, surface micro inspection is a necessary method for analyzing the contact state and material damage mechanism. In this study, the non-uniform characteristic of corrugated damage was given special attention. Under optical microscope, scanning electron microscope and white light interference microscope, the morphology corresponding to the “light” and “dark” area were distinguished. The distribution of the composition and hardness of the corrugated area were characterized in detail. Finally, a possible mechanism was proposed to attempt to explain the formation of corrugated damage. The results of this article may be helpful for a deeper understanding of corrugated damage of bearing in electric field environments, and could shed new light on the research on lubricated current-carrying tribology.

## 2. Experimental Method

### 2.1. Test Method for Electrical Erosion of Bearings

**Figure 1:**
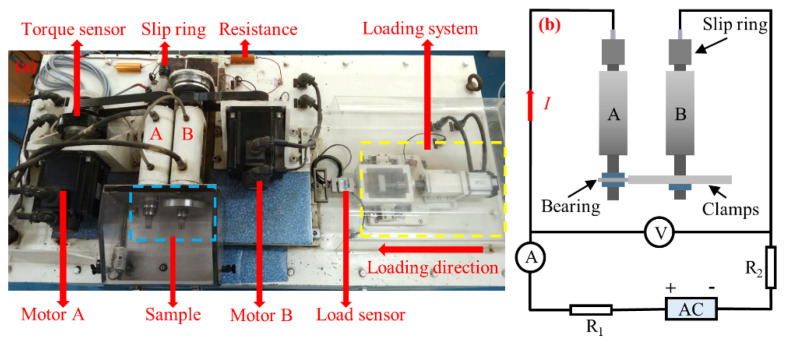
Schematic diagram of the test equipment. (**a**) Rolling current-carrying tribometer; (**b**) schematic diagram of the current-carrying system.

A rolling current-carrying tribometer (FTM-CF100, Nanjing Bio-Inspired Technology Co. Ltd., Nanjing, China) was used to test the electrical erosion of the bearing. The main structure of the equipment included a loading system, spindles A and B, a torque sensor, and a current-carrying system (Figure 1a).

The bearing samples and clamps are mounted on spindles A and B, respectively. Spindle A is driven by motor A, which drives the bearing sample to rotate. Spindle B and its driving motor B are installed on the displacement table, which is pushed by the loading system and approaching spindle A. Spindle B is fixed and does not rotate under the action of a brake system. A load sensor is used to measure the contact load *F* (when the load reaches 1000 N, the motor automatically stops to protect the sensor from damage). When the contact load between the bearing and clamps reaches the preset value, the loading system stops running. Before each test, the torque sensor mounted on motor A should be calibrated to zero so that the measured torque value *T* during the rolling comes from bearing friction *f*. The friction coefficient *μ* can be calculated as (1) and (2):

(1) $$f=\frac{2T}{D}$$

(2) $$\mu =\frac{2T}{DF}$$

In the current-carrying system (Figure 1b), the current *I* supplied by the AC power passes through the resistance *R*_1_, slip ring, spindle A, bearing, clamps, spindle B, and resistance *R*_2_ such that the bearing is in a conductive state during the test. Based on the voltage drop *U_d_* between axes A and B and the current *I*, the equivalent resistance of bearing *R_e_* can be calculated as (3)

(3) $$R_{e}=\frac{U_{d}}{I}-R_{s}$$

*R_s_* = 20 mω is the sum of static resistances of slip rings, spindle A, clamps, spindle B and wires.

The sample is a 61,808 deep groove ball bearing, internally filled with lithium grease for good resistance to oxidation, erosion, and water. The geometrical characteristics of the bearing sample are listed in Table 1. The chemical composition (in wt.%) of the bearing material (GCr15) is listed in Table 2.

To explore the formation process of the corrugated damage, the AC voltage between rotating axes A and B was set as 0, 1.2, 2.2, 2.7, 3.2, 3.7, and 4.2 V, respectively, and the AC frequency was 400 Hz. This voltage amplitude is within the range of shaft voltage in the engineering field [3]. The speed of rotating Spindle A was 600 r/min, and the normal load was 100 N. The setting of the rotational speed is in accordance with the typical application of 61,808 bearings such as the motors of pumps, lathes, and welding machines. Based on the geometry of the bearing and the number of balls, the maximum Hertzian contact pressure between ball and races is calculated as 1281.49 MPa (inner race) and 1268.85 MPa (outer race) [13]. The yield strength of the GCr15 bearing steel is higher than 2000 MPa after quenching and tempering heat treatment [16], so that the bearing is in elastic contact in this paper. Spindle B does not rotate and only provides static contact with the outer raceway. To calculate the rate of grease leakage, the bearings were weighed by the electronic analytical balance (FA224C, Lichen, Shanghai, China) with accuracy of 0.1 mg before and after the test. An infrared thermometer (Smart Sensor AS852B, SMART SENSOR, Shenzhen, China) was used to measure the temperature increase in the outer ring of the bearings. The test of each group lasted for 2 h and each experiment was repeated three times.

### 2.2. Surface Analysis

In this experiment, corrugated damage was only found on the outer ring; thus, a more detailed surface analysis was conducted on the outer ring.

After the test, the grease was cleaned, and the outer race was cut into pieces using an EDM CNC wire-cutting machine (DK7735, Weihan CNC, Suzhou, China). To distinguish the details of the surface damage, the outer race sample and balls were observed using optical microscope (OM, Leica DMi8C, Mannheim, Germany), scanning electron microscope (SEM, JSM-5610LV, Tokyo, Japan), and a white light interference 3D profiler (WLIP, µSurf, NanoFocus AG, Oberhausen, Germany). The outer raceway was cut along the rolling direction to observe the effect of electrical erosion on the material structure, which exposed the subsurface of the contact area. After cutting, the samples were polished on an XQ-2B polishing machine (Shenzhen Rongda Instrument Equipment Co., Ltd., Shenzhen, China) and then were etched with 4% nitric acid alcohol solution for 9 s. Metallographic structures were observed using an optical microscope. The hardness value of the contact surface and that in the depth direction beneath the contact area were measured using a microhardness tester (HV-1000, Zhongte Technology, Dongguan, China) under an applied load of 200 gf. To obtain the residual compressive stress, an X-ray residual stress analyzer (LXRD, Proto Manufacturing Ltd., LaSalle, ON, Canada) was used to scan the outer race. All the samples were ultrasonically washed in anhydrous ethanol for 15 min prior to characterization.

## 3. Results and Discussion

### 3.1. Effect of Voltage Amplitude on Current-Carrying Tribological Performance

**Figure 2:**
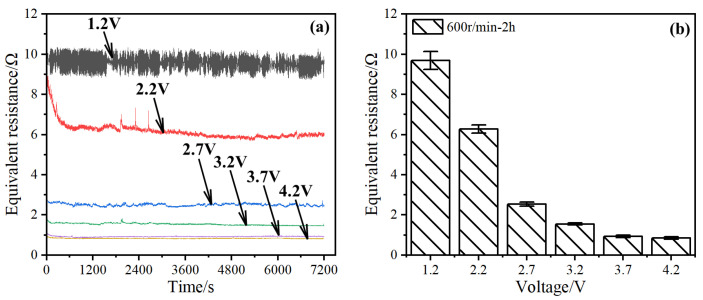
Equivalent resistance of bearings at different voltage amplitudes. (**a**) Real-time equivalent resistance; (**b**) average equivalent resistance at 6000–7200 s stabilization stage.

The equivalent resistances of the bearings at different voltages are shown in Figure 2.

When the voltage was 1.2 V, the equivalent resistance curve exhibits severe fluctuations (Figure 2a). As the voltage increased, the fluctuation in the equivalent resistance decreased, and the value of the entire curve decreased. When the voltage was 2.7 V, no evident burrs were observed in the equivalent resistance curve. When the voltage increased to 3.7 and 4.2 V, the two curves almost coincided and were close to the shape of a straight line. Based on the resistance curves between 6000 and 7200 s, the average value decreased with increasing voltage and eventually stabilized (Figure 2b). The average contact resistance of the bearing reached the lowest at 3.7 and 4.2 V, which was only 0.8 Ω.

It was reported that an insulating oil film with a thickness of 0.1 to 1.4 μm usually formed inside the bearing, which was equivalent to a complex capacitor system after the application of electricity [4]. The oil film acts as a dielectric medium and the balls and raceways act as capacitor plates [17]. The capacitor is discharged when the voltage reaches the breakdown limit of the oil film [18]. At lower voltages, the partial oil film underwent breakdown. As the voltage increased, the degree of breakdown increased, indicating that there were more channels for direct current flow, resulting in a decrease in the equivalent resistance. After the complete breakdown of the oil film, the capacitive contact of the bearing changed to a resistive contact; therefore, the resistance did not change after the breakdown [12]. The bearing breakdown voltage under the test conditions used in this study is 3.7 V.

**Figure 3:**
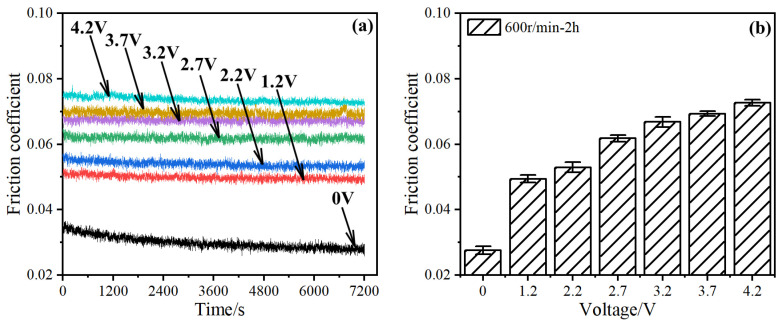
Friction coefficients of bearings at different voltage amplitudes. (**a**) Real-time friction coefficient; (**b**) average friction coefficient at 6000–7200 s stabilization stage.

Figure 3 shows the friction coefficient of the bearing at different voltage amplitudes. Without an applied voltage, the friction coefficient slowly decreased and eventually stabilized (Figure 3a), and the average value was only 0.028 during the stable stage (6000–7200 s).

As the voltage increased from 0 to 4.2 V, the average friction coefficient at the stable stage increased from 0.028 to 0.073. The results indicated that the voltage was harmful to the formation of the lubrication film [19].

**Figure 4:**
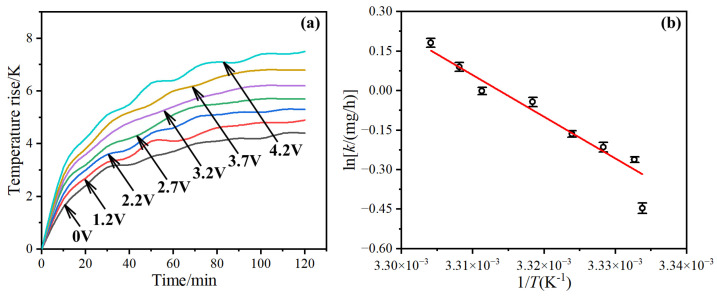
The temperature rise of outer ring (**a**) and the rate of lubrication leakage (**b**) under different voltage amplitudes.

The temperature increase in the outer ring of the bearings was measured to find out the effect of voltage on friction performance of the bearings. After 100 min of testing, the temperature rise was basically stable, and as the voltage increased from 0 to 4.2 V, the temperature rise of the outer ring increased from 4.4 to 7.5 K (Figure 4a).

Lubrication leakage was also observed during the test, and the bearing weight before and after the test was determined to obtain the lubrication weight loss. An increase in temperature causes a decrease in grease viscosity [20], leading to easier leakage. After fitting, the average rate of lubrication leakage satisfied the Arrhenius equation [21,22]:

(4) $$\ln\;k\;=\frac{{-E}_{a}}{RT}+\ln\;A\;$$

*k*—Rate of lubrication leakage at a temperature *T*

A—Arrhenius constant

*E_a_* —Activation energy of the experiment

R—Molar gas constant

*T*—Absolute temperature

The fitting result in Figure 4b showed that *E_a_* = 346.37 kJ/mol.

Lubrication leakage and lower viscosity indicate a decrease in lubricant film thickness. In addition, a temperature increase can accelerate lubrication aging, and the transient high temperature of breakdown can cause grease carbonization [23,24,25]. These factors can lead to an increase in the friction coefficient.

### 3.2. The Effect of Voltage Amplitude on the Formation of Corrugated Damage

**Figure 5:**
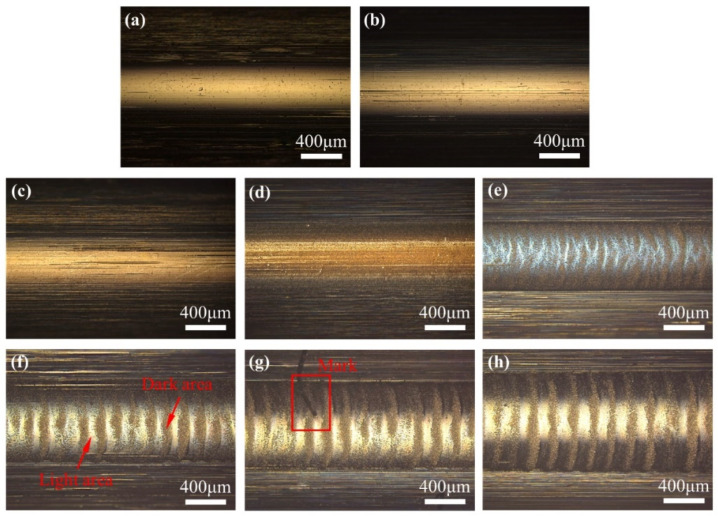
Optical microscope morphology of outer race surface (×50); (**a**) Original; (**b**) 0 V; (**c**) 1.2 V; (**d**) 2.2 V; (**e**) 2.7 V; (**f**) 3.2 V; (**g**) 3.7 V; (**h**) 4.2 V.

After the test, the bearing was cut into pieces and the grease was removed. The outer raceways and balls were observed using an optical microscope. The surface of the original outer raceway, shown in Figure 5a, was smooth and showed no wear damage.

After mechanical rolling, a few furrows appeared on the raceway surface (Figure 5b). Under 1.2 and 2.2 V, the furrows on the race surface increased and dense electrical erosion pits appeared (Figure 5c,d). Corrugations were observed on the raceway at 2.7 V; the boundary between the light and dark areas was unclear. When the voltage increased to 3.2, 3.7, and 4.2 V, evident corrugated damage was formed. The light and dark areas of the damaged morphology are shown in Figure 5f.

Corrugated damage has attracted the attention of researchers for a long time, and the representation of this damage has mostly been in terms of lightness and darkness. However, more detailed differences, such as wear depth, composition, hardness, and damage mechanism, have not been studied systematically. Furthermore, the light and dark states of the damaged area obtained using the different imaging methods were not the same, which could easily result in confusion. Therefore, a mark was made on the surface, as shown in Figure 5g, to locate the damaged area.

**Figure 6:**
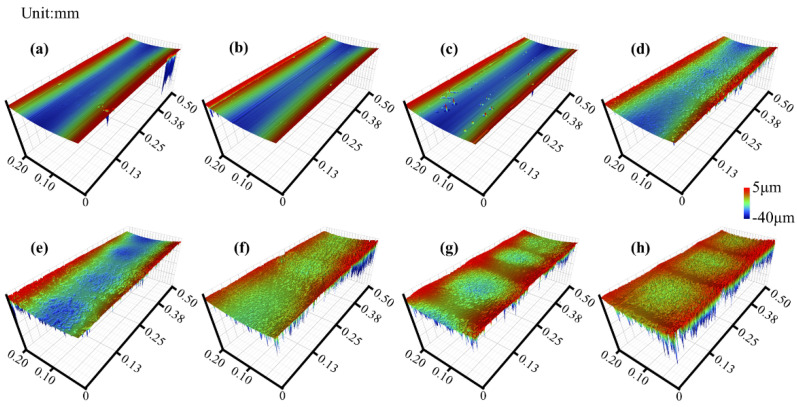
Three-dimensional morphology of outer race surface; (**a**) original; (**b**) 0 V; (**c**) 1.2 V; (**d**) 2.2 V; (**e**) 2.7 V; (**f**) 3.2 V; (**g**) 3.7 V; (**h**) 4.2 V.

The corresponding 3D morphologies of the samples are shown in Figure 6, and the morphology information of the worn surface is summarized in Table 3.

The surface roughness of the original raceway surface was 0.59 μm, and increased from 0.70 to 1.64 μm after rolling as the voltage increased from 0 to 4.2 V. On the surface of the 1.2 and 2.2 V samples, some randomly distributed pits and scratches along the rolling direction were found, and the surface damage mechanisms were electrical erosion and abrasive wear. Corrugated damage started to form on the surface of the 2.7 V sample. As the voltage increased, the shape of the corrugated damage became more evident. On the sample at 2.7 V, the difference in height between the light and dark areas was 0.30 μm. When the voltage was increased to 4.2 V, the difference in height between the light and dark areas increased to 2.66 μm. Based on the positioning of the scratch mark in Figure 5g, the high area in the 3D morphology is the light area in the image obtained using optical microscopy (in Supplementary Materials).

**Figure 7:**
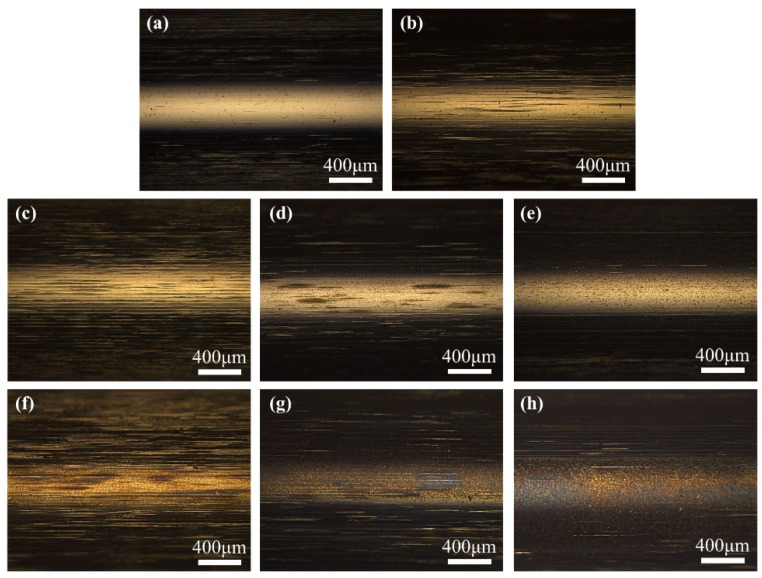
Optical microscope morphology of inner race surface (×50); (**a**) original; (**b**) 0 V; (**c**) 1.2 V; (**d**) 2.2 V; (**e**) 2.7 V; (**f**) 3.2 V; (**g**) 3.7 V; (**h**) 4.2 V.

Figure 7 shows the results of optical microscopy of the bearing inner ring under different working conditions. The surface of the original samples was smooth and clean, after mechanical rolling, furrows began to appear on the surface of the samples. A small number of pits and furrows existed on the surface of the 2.2 V samples; the wear mechanisms comprised erosion wear and abrasive wear. As the voltage increased, the inner ring electrical erosion became more severe, but no corrugated damage was found in any of the samples.

**Figure 8:**
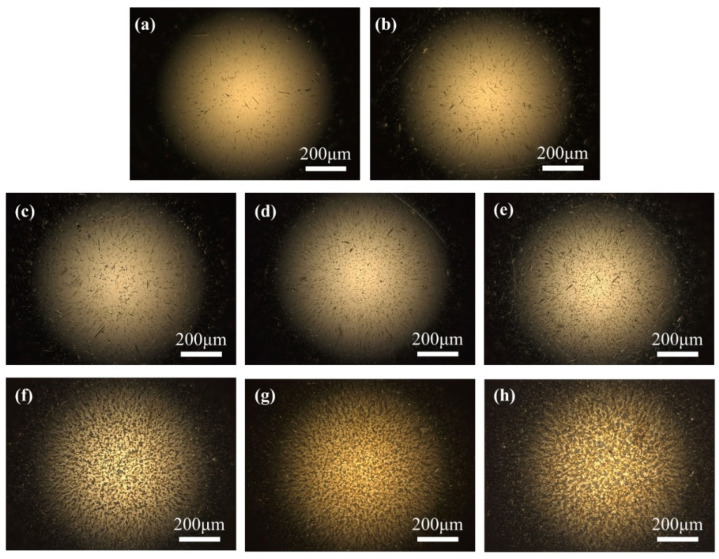
Optical microscope morphology of bearing ball (×100); (**a**) original; (**b**) 0 V; (**c**) 1.2 V; (**d**) 2.2 V; (**e**) 2.7 V; (**f**) 3.2 V; (**g**) 3.7 V; (**h**) 4.2 V.

Figure 8 shows the results of optical microscopy of the bearing balls under different working conditions.

No corrugated damage was observed, but dense pits were formed on the surface of the balls. The higher the voltage, the more severe was the electrical erosion of the ball. The difference between the ball and inner and outer raceway may be related to the mode of movement. The inner raceway rotated with the axes of rotation, the rolling and spinning of the balls made the surface damage more uniform [26] and caused less periodic damage. When the outer raceway was relatively fixed, the contact state was unchanged, and it was easier to form regular damage. There are no flat wear areas or deep grooves formed on the rollers, making it impossible to obtain the wear depth or wear volume of the rollers.

**Figure 9:**
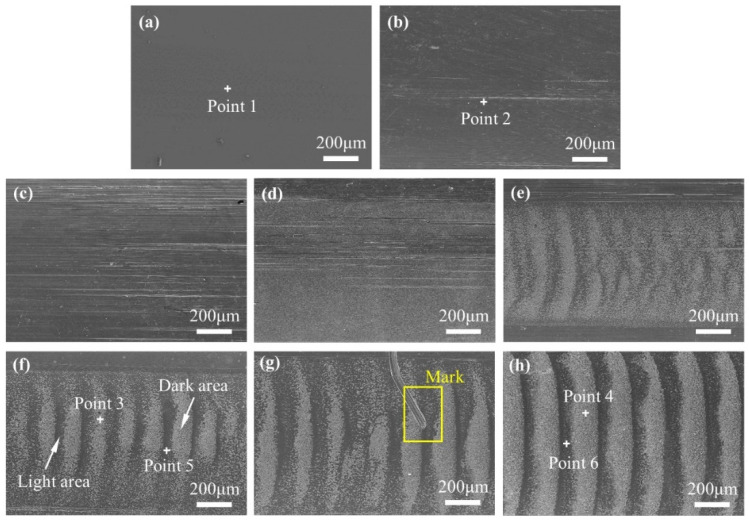
SEM images of outer race surface of bearing; (**a**) original; (**b**) 0 V; (**c**) 1.2 V; (**d**) 2.2 V; (**e**) 2.7 V; (**f**) 3.2 V; (**g**) 3.7 V; (**h**) 4.2 V.

SEM images of the raceway surface were obtained to further understand the morphological characteristics of the bearing damage (Figure 9).

Figure 9a shows that the original raceway surface of the sample was smooth and clean. A small number of furrows appeared on the raceway surface after mechanical rolling (Figure 9b). By applying 1.2 V to the bearing, a significant increase in the number of surface scratches was observed (Figure 9c). Randomly distributed electrical erosion pits were observed when the voltage increased to 2.2 V (Figure 9d), indicating that the lubricating film was partially broken down. As the voltage continued to increase, the surface erosion became more severe [27], and irregular corrugated damage appeared on the surface at 2.7 V voltage (Figure 9e). When the voltage was higher than 3.2 V, clear corrugated damage was formed on the surface (Figure 9f–h). Figure 9g shows a scratch mark adjacent to the wear area. By comparing the OM, 3D, and SEM images, it can be observed that the light area in the optical microscope appears dark in the SEM image.

**Figure 10:**
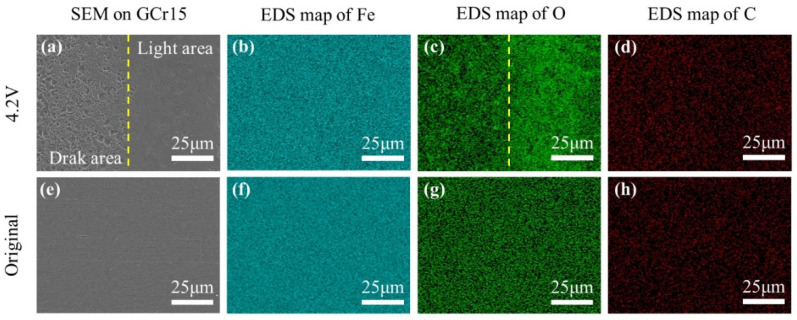
EDS maps of outer race surface of bearing; (**a**–**d**) corrugated damage area on the sample at 4.2 V; (**e**–**h**) original sample.

The light-dark boundary in Figure 9h was selected for a more detailed observation. Figure 10a shows that a large number of ablation pits were formed in the dark area, and EDS showed that the oxygen content of the dark area was lower than that of the light area, while Fe and C were uniformly distributed (Figure 10b–d).

The distributions of Fe, C, and O on the original sample surface were uniform and are shown in (Figure 10f–h) for comparison. Generally, the arc ablation area is much hotter and more prone to oxidation [28]. It is speculated that oxidation was inhibited in the electric ablation area (dark area).

**Figure 11:**
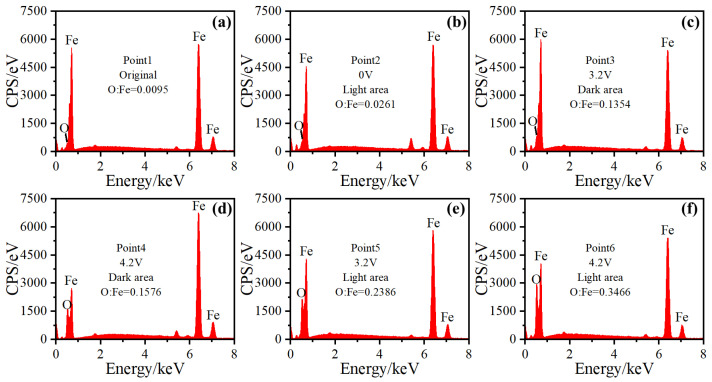
O:Fe atomic ratio at different locations of corrugated damage area. (**a**) Point 1 in Figure 9a; (**b**) Point 2 in Figure 9b; (**c**) Point 3 in Figure 9f; (**d**) Point 4 in Figure 9h; (**e**) Point 5 in Figure 9f; (**f**) Point 6 in Figure 9h.

To further confirm the non-uniformity of the oxidation, energy spectrum signals were collected at different locations, as shown in Figure 9. The original raceway is protected with grease. and the O:Fe atomic ratio is only 0.0095 (Figure 11a).

The raceways were still protected by grease during the mechanical rolling, and the O:Fe atomic ratio increased slightly to 0.0261 (Figure 11b). As the voltage increased to 3.2 V and 4.2 V, the degree of bearing breakdown increased and there was evident electrical erosion on the bearing surface. The O:Fe atomic ratio in the corrugated damaged area increased intensely in both the dark and light areas. The degree of oxidation in the dark areas (Figure 11c,d) was lower than that in the light areas (Figure 11e,f). The results of EDS surface scanning and point scanning showed that the degree of oxidation of the dark area with dense electrical erosion was lower.

The above analyses showed that the corrugated damage was nonuniform, with differences in morphology and composition between the light and dark areas. The dark areas under the OM appeared as light areas under the SEM with the help of scratch mark positioning. The electrical erosion damage in the dark area was more intensive, and thus, material removal was more evident. The increase in the voltage was beneficial for the formation of corrugated damage. Under the experimental conditions, the corrugated damage could be produced when the voltage reached 73% of the complete breakdown voltage. The differences in the damage morphology and degree of oxidation indicated that the damage mechanisms in the light and dark regions were different.

### 3.3. Effect of Voltage Amplitude on the Microstructural Changes

**Figure 12:**
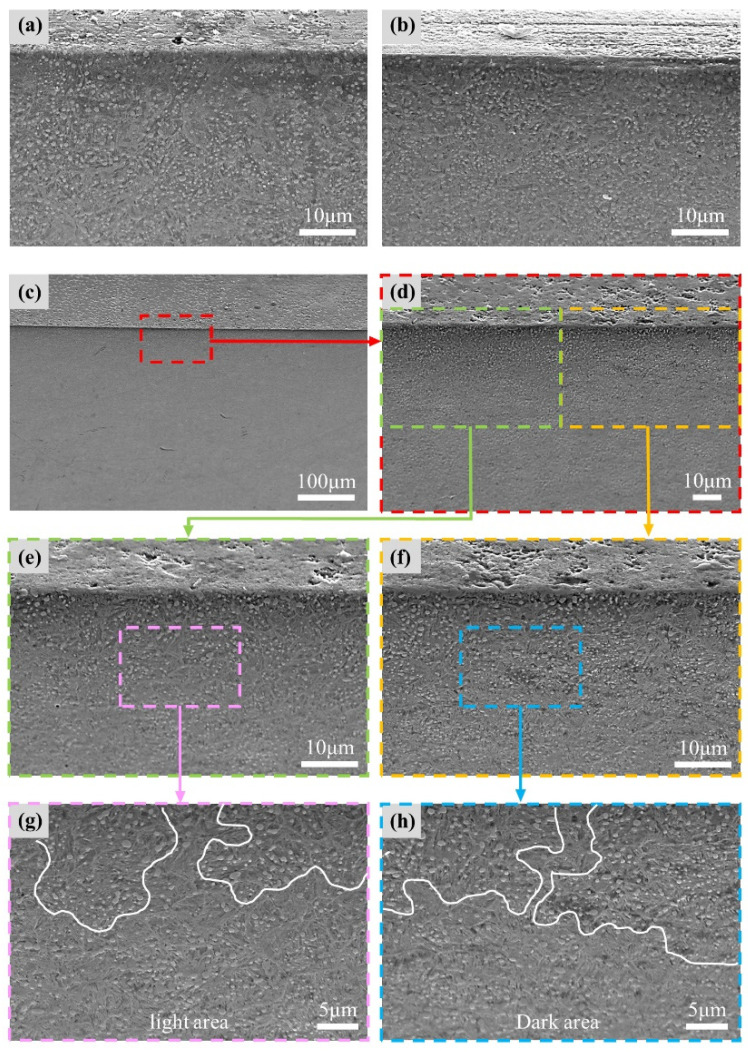
SEM image of the microstructures beneath the outer raceways after rolling at different voltages; (**a**) original (×2000); (**b**) 2.2 V (×2000); (**c**) 4.2 V (×200); (**d**) 4.2 V (×1000); (**e**) light area in 4.2 V sample (×2000); (**f**) dark area in 4.2 V sample (×2000); (**g**) light area in 4.2 V sample (×3000); (**h**) dark area in 4.2 V sample (×3000).

During rolling, the bearing steel may undergo structural changes due to mechanical and heating effects [29,30]. The average temperature rise of the bearing samples under different voltage conditions is presented in Figure 4, which is lower than the low-temperature tempering temperature of the bearing steel and is insufficient to change the microstructure of the bearing steel. However, the high temperature of the arc discharge can reach 7000–8000 K [31], which is much higher than the melting point of the GCr15 bearing steel. To observe the microstructural changes induced by discharge, the bearing samples were cut along the raceway and etched with 4% nitric acid ethanol solution for approximately 9 s. After cleaning and drying, the microstructure was observed through SEM (Figure 12).

The original structure of the bearing steel was fine martensite with uniformly distributed carbides (Figure 12a). At 2.2 V, the rolling surface undergoes electrical erosion (Figure 9d); however, no structural changes are observed in the subsurface layers (Figure 12b). As shown in Figure 12c,d, the corrugated damage was positioned on the surface at 4.2 V. Carbides are enriched in the subsurface layer (Figure 12d). By observing the light and dark areas separately, the phenomenon of carbide enrichment became clearer (Figure 12e,f). At higher magnification, it could be observed that the enrichment degree of carbide in dark area (Figure 12h) was higher than that in light area (Figure 12g). Based on a comparison of the surface morphologies (Figure 9 and Figure 10) and subsurface structures (Figure 12), the degree of carbide enrichment was related to the degree of electrical erosion. During discharge, the transient high temperature caused melting and splashing, forming erosion pits. The high temperature around the discharge position caused the decomposition of martensite in the heat-affected zone. Supersaturated C atoms precipitated from martensite and combined with metal Fe to form carbides [32]. Because of the extremely short discharge process, the heat-affected zone was concentrated in the subsurface, resulting in the enrichment of carbides.

**Figure 13:**
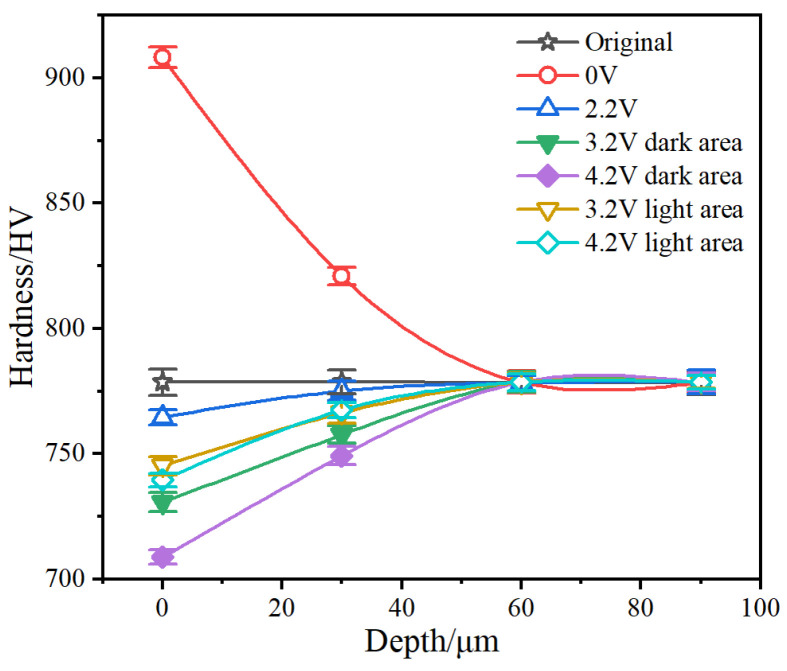
The hardness beneath the wear surface of outer raceway.

The mechanical performance of the steel was closely related to its microstructural changes. To analyze the effect of corrugated damage at different voltages on mechanical performance, the hardness of the subsurface of the worn-out samples was tested along the depth direction (Figure 13).

The hardness of the original sample was uniform, with a value of 778.6 HV from the surface to a depth of 90 μm. After mechanical rolling at 600 r/min for 2 h, a work-hardening effect was [22] observed, and the surface hardness increased to 908.3 HV. This work hardening may be related to the proliferation of superficial dislocations and stress-induced martensitic phase transformation [22]. The hardness recovered to 778.6 HV when the depth was increased to 60 μm. At a voltage of 2.2 V, the hardness was 764.6, 775.1, 778.6, and 778.6 HV at 0, 30, 60, and 90 μm depth, respectively. At a voltage of 3.2 V, as the depth increased from 0 to 60 μm, the hardness of the light area decreased from 778.6 to 745.2 HV, and the hardness of the dark area decreased from 778.6 to 730.6 HV. The hardness of the light area is slightly higher than that of the dark area at the same depth. When the voltage was 4.2 V, the hardness of the light area decreased from 778.6 to 739.8 HV, and the hardness of the dark area decreased from 778.6 to 708.8 HV. The difference between the hardness values of the light and dark areas increased. Therefore, the more severe the electrical erosion, the lower the hardness. The hardness of the dark area was lower than that of the light area owing to the nonuniform erosion in the corrugated damage.

**Figure 14:**
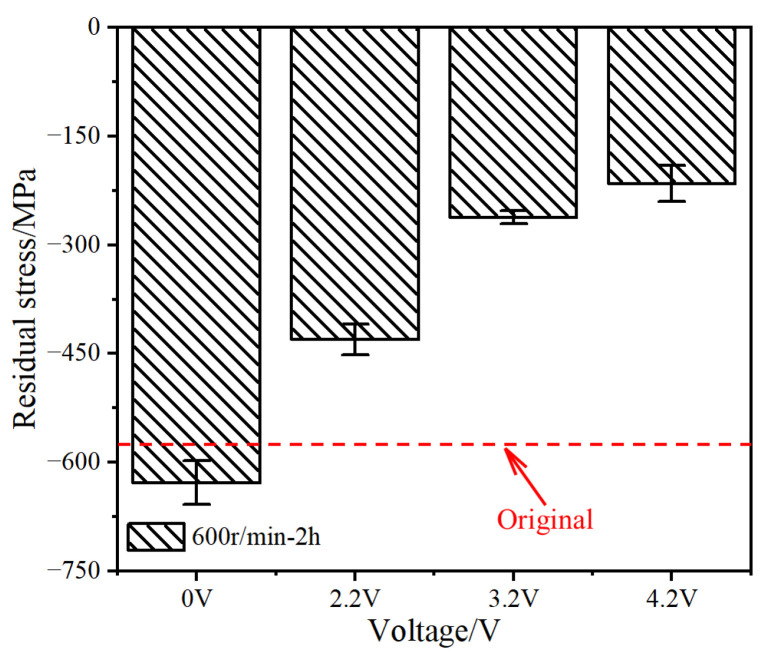
Residual stress of outer raceway.

The bearing material hardness can also be characterized using internal stresses [33]. Figure 14 shows the internal stresses on the surfaces of different samples.

Negative stress indicates the residual compressive stress; the higher the stress value, the greater the hardness. The residual compressive stress of the original sample was 570.01 MPa. After pure mechanical rolling, the residual stress increased to 628.27 MPa. When the voltage applied to the bearing increased from 2.2 to 4.2 V, the residual compressive stress decreased from 430.46 to 215.27 MPa. Owing to the limited resolution of the X-ray residual stress analyzer, it was not possible to identify independent stresses in the dark and light areas. Nevertheless, the results shown in Figure 14 indicate that electrical erosion reduces the mechanical performance of the bearing.

### 3.4. The Possible Formation Mechanism of Corrugated Damage

The formation of corrugated damage is closely related to bearing breakdown. The morphology and composition results showed that a partial breakdown could only cause a disorderly distribution of erosion pits (Figure 9d). When the breakdown reaches a certain degree, corrugated damage begins to appear. Based on the analysis of the morphology, composition, and hardness, the light and dark areas should undergo different damage processes. Only when a stable lubricating film is established in the bearing, the contact area can form a metal-insulation film–metal capacitive structure, which may break down and cause electrical erosion [34,35]. When the lubrication is destroyed, the lubricating film adsorbed on the surface of the bearing steel is extremely thin, and the metal–metal resistance structure can dominate the contact area. Current may flow directly through the resistance, making it difficult to generate a breakdown.

**Figure 15:**
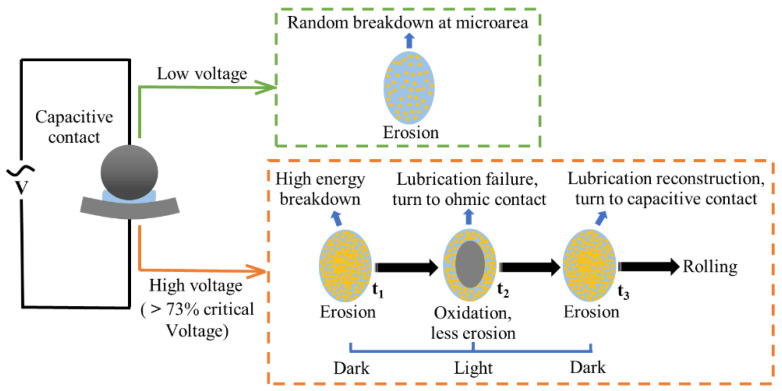
Schematic diagram of the formation of corrugated damage.

As shown in Figure 15, a corrugated damage formation mechanism is proposed to explain the phenomenon observed in this study.

A metal-insulation film–metal capacitive structure was established after the bearing was rotated. When the applied voltage was low, only localized breakdown occurred at the position where the oil film was thin on the rough surface. Randomly distributed erosion pits were formed on the bearing surface. When the voltage reached a certain level (higher than 73% of critical voltage), the process of the formation of the corrugated damage may be as follows:

i The discharge energy was high enough to breakdown the lubrication film, and a large number of erosion pits were formed (Figure 15, *t*_1_). Electrical erosion dominants the surface damage
ii Then, the lubrication film could be pushed out of the contact area owing to high-temperature vaporization, an ohmic contact could form, and the current could directly pass through the contact area, decreasing the degree of erosion (Figure 15, *t_2_*). Because of the loss of lubrication coverage, oxygen in the atmosphere could come into contact with the metal, causing oxidation. Wear and oxidation dominants the surface damage.
iii As the roller continued to move forward, the surrounding grease filled the contact area, and the lubrication film was re-established, inducing the next round of erosion (Figure 15, *t*_3_).

During the cycle of destruction and reconstruction of the lubrication film, the conductive state of the contact area undergoes periodic changes, so that the form of bearing damage is also periodic. Erosion in the dark area was more severe, resulting in a poorer surface quality. The rougher contact in the dark area was more likely to cause the next round of discharge. Once subsequent breakdown occurs selectively and preferentially in the electro dark area, the boundary between the light and dark areas of damage can become clearer and clearer. In this manner, a corrugated damage with alternating light and dark contrasts was formed. We attempted to reveal the formation mechanism of corrugated damage and provided a reasonable explanation for the differences in morphology, hardness, oxidation, and residual stress in the damaged area. However, it is still impossible to answer why there is no corrugated damage on the inner raceway and ball bearings. In this experiment, the inner raceway and ball bearings were always in rolling motion, and the contact between the two was random, resulting in a uniform distribution of surface damage. After comparison, it can be concluded that corrugated damage is more likely to form on fixed pair. The outer ring is fixed, and the contact state is regularly distributed along the raceway. However, it is currently impossible to speculate on the formation process of corrugated damage. Further exploration is needed to determine whether corrugated damage is related to the distribution of contact states.

The Bearing breakdown level was sufficiently high, and the bearing was in an ohmic contact state before lubrication reconstruction, which is a necessary condition for the formation of corrugated damage. The nonuniformity of the corrugated damage indicates periodic changes in the electrical contact state of the bearing. However, there are still certain issues that require further exploration, such as the dynamic formation process of corrugated damage, the relationship between corrugated damage and contact states, the optimal location for the formation of corrugated damage on the raceway, the relationship between the periodicity of corrugated damage and AC frequency, and the relationship between corrugated damage and vibration. Preventing breakdown is an effective way to suppress bearing electrical erosion. For example, the insulation coating on the inner and outer rings can reduce shaft current, while conductive grease and grounding can reduce shaft voltage. However, due to the presence of leakage current, bearing erosion cannot be completely eliminated. The use of ceramic rolling elements can prevent bearing breakdown, but due to factors such as low impact resistance, they have not been widely used. With the increase in bus voltage and speed, bearing erosion remains an urgent problem in high-speed trains, new energy vehicles, wind power and other fields. It is necessary to further explore the mechanism of bearing erosion and develop bearing erosion protection technologies.

## 4. Conclusions

Corrugated damage on 61,808 bearings under different amplitude AC voltages were studied, and the non-uniform damage mechanism was analyzed based on the morphology, surface composition, subsurface microstructure, hardness, and residual stress. The conclusions are summarized as follows:

1. As the voltage applied to the bearing increased from 0 V to 4.2 V, the equivalent resistance decreased, and the friction coefficient increased. The temperature rise of the outer ring slowly rises but eventually stabilizes after 100 min. The average rate of lubrication leakage satisfies the Arrhenius equation. The critical breakdown voltage of the bearing was 3.7 V under these experimental conditions.
2. Corrugated damage could be produced in the outer race when the voltage reached 73% of the complete breakdown voltage. The light area in the images obtained using optical microscope and three-dimensional profiler appeared dark in those by a scanning electron microscope. Greater erosion, lower oxidation, and lower height were observed in the dark area. However, the erosion pits were evenly distributed on the balls and inner race.
3. The tempering effect of the discharge caused decomposition of the subsurface martensite, leading to the enrichment of carbides on the subsurface, a decrease in residual stress hardness. When the voltage applied to the bearing increased from 2.2 to 4.2 V, the residual compressive stress decreased from 430.46 to 215.27 MPa after rolling, which was lower than the original 570.01 MPa. As a comparison, the residual stress increased to 628.27 MPa after mechanical rolling.
4. A possible formation mechanism for corrugated damage was proposed to explain the experimental phenomenon. Under capacitive contact, a sufficiently high voltage (>73% of the complete breakdown voltage) not only caused metal erosion of the bearing, but also destroyed the bearing lubrication film. Then, the capacitive contact transformed into a resistive contact, reducing electrical erosion but intensifying oxidation. Subsequently, the lubricating film was reconstructed during rolling, inducing the next round of electrical corrosion. The periodic characteristics of corrugated damage may be related to the cyclic switching between capacitive contact and resistive contact in bearings.

## Figures and Tables

**Table 1:** The geometrical characteristics of the bearing sample.

Parameters	Values
Outer diameter *D*/mm	52
Inner diameter *d*/mm	40
Thickness B/mm	7
Ball diameter/mm	3.14
Number of bearing balls	24
Weight/g	About 34
Limiting speed (r/min)	16,000
Rated dynamic load/N	4200
Rated static load/N	4950
Fatigue limit load /N	186

**Table 2:** The chemical composition (in wt.%) of the bearing material.

Elements	C	Mn	Si	Cr	Mo	Ni	Fe
Contents	0.95–1.05	0.20–0.40	0.15–0.35	1.30–1.65	≤0.10	≤0.30	Bal.

**Table 3:** The morphology information of the worn surface.

Test Conditions	Original	0 V	1.2 V	2.2 V	2.7 V	3.2 V	3.7 V	4.2 V
Width of light area/μm	/	/	/	/	36.16 ± 2.42	41.62 ± 4.79	44.17 ± 4.28	46.50 ± 6.36
Width of dark area/μm	/	/	/	/	89.97 ± 2.19	98.50 ± 5.81	105.54 ± 6.25	139.02 ± 6.74
Height difference between corrugation/μm	/	/	/	/	0.30 ± 0.07	1.26 ± 0.41	1.39 ± 0.57	2.66 ± 0.72
Surface roughness Ra/μm	0.59 ± 0.15	0.70 ± 0.18	0.72 ± 0.16	0.80 ± 0.18	0.81 ± 0.13	1.05 ± 0.11	1.09 ± 0.25	1.64 ± 0.28

## Data Availability

The original contributions presented in the study are included in the article/supplementary material, further inquiries can be directed to the corresponding author.

## Supplementary Materials

The following supporting information can be downloaded at: https://www.mdpi.com/article/10.3390/ma17040859/s1, Figure S1: 3D morphology of outer race surface. (a) Original; (b) 0 V; (c) 1.2 V; (d) 2.2 V; (e) 2.7 V; (f) 3.2 V; (g) 3.7 V; (h) 4.2 V.

## References

[1] Turnbull R., Rahmani R., Paul S., Rahnejat H. (2023). Electrotribodynamics of ball bearings in electrical machines. Tribol. Int..

[2] Vostrov K., Pyrhonen J., Niemela M., Lindh P., Ahola J. (2023). On the Application of Extended Grounded Slot Electrodes to Reduce Noncirculating Bearing Currents. IEEE Trans. Ind. Electron..

[3] Berhausen S., Jarek T. (2021). Method of Limiting Shaft Voltages in AC Electric Machines. Energies.

[4] He F., Xie G., Luo J. (2020). Electrical bearing failures in electric vehicles. Friction.

[5] Teshale A., Biru G. (2023). Overview of shaft voltage and bearing current mitigation methods applied on the victim machine. Electr. Eng..

[6] Liu W. (2014). The prevalent motor bearing premature failures due to the high frequency electric current passage. Eng. Fail. Anal..

[7] Zika T., Gebeshuber I.C., Buschbeck F., Preisinger G., Gröschl M. (2009). Surface analysis on rolling bearings after exposure to defined electric stress. Proc. Inst. Mech. Eng. Part J J. Eng. Tribol..

[8] Ma J., Xue Y., Han Q., Li X., Yu C. (2022). Motor Bearing Damage Induced by Bearing Current: A Review. Machines.

[9] Oliver J.A., Guerrero G., Goldman J. (2017). Ceramic Bearings for Electric Motors: Eliminating Damage with New Materials. IEEE Ind. Appl. Mag..

[10] Kudelina K., Vaimann T., Rassolkin A., Kallaste A., Asad B., Demidova G. Induction Motor Bearing Currents—Causes and Damages. Proceedings of the 2021 28th International Workshop on Electric Drives: Improving Reliability of Electric Drives (IWED).

[11] Liu Z., Song C., Li J., Hou X., Wang L., Zhang Y. (2020). Effect of Rotation Speed on Electric Damage of Copper Tribo/Electric Rolling Pairs under Lubricated Single-Point Contact. Mater. Trans..

[12] Niu K., Song C., Lou Z., Pang X., Lu H., Du S., Zhang Y. (2023). Electric damage of bearing under AC shaft voltage at different rotation speeds. Tribol. Int..

[13] Chen T., Song C., Liu Z., Wang L., Sun C., Pang X., Zhang Y. (2023). Effect of Elastic Contact Force on Tribological Characteristics of Current-carrying Roll Rings in Rotating Conductive Joints. Tribol. Trans..

[14] Seferi Y., Blair S.M., Mester C., Stewart B.G. (2021). A Novel Arc Detection Method for DC Railway Systems. Energies.

[15] Derosa S., Nåvik P., Collina A., Bucca G., Rønnquist A. (2020). A heuristic wear model for the contact strip and contact wire in pantograph—Catenary interaction for railway operations under 15 kV 16.67 Hz AC systems. Wear.

[16] Li N., Cui C., Zhao Y., Zhang Q., Bai L. (2018). Structure and properties of GCr15 modified by multiphase ceramic nanoparticles /Fe-C composite inoculants. Mater. Sci. Eng. A.

[17] Ren X., Liu R., Yang E. (2019). Modelling of the bearing breakdown resistance in bearing currents problem of AC motors. Energies.

[18] Romanenko A., Muetze A., Ahola J. (2016). Effects of electrostatic discharges on bearing grease dielectric strength and composition. IEEE Trans. Ind. Appl..

[19] Hanrahan B., Misra S., Waits C.M., Ghodssi R. (2015). Wear mechanisms in microfabricated ball bearing systems. Wear.

[20] Yang S., Li C., Jiang M., Pei S. (2019). A study of inlet temperature models of a large size tilting thrust bearing comparison between theory and experiment. Tribol. Int..

[21] Lugt P.M. (2023). On the use of the Arrhenius equation to describe the impact of temperature on grease life. Tribol. Int..

[22] Ma Z.-M., Zhu H., Cao Y.-B., Yang S.-P. (2020). Evolution of microstructure and mechanical properties of the high-speed train bearing under different service periods. Mater. Sci. Eng. A.

[23] Lugt P.M. (2009). A review on grease lubrication in rolling bearings. Tribol. Trans..

[24] Romanenko A., Ahola J., Muetze A. (2015). Influence of electric discharge activity on bearing lubricating grease degradation. Proceedings of the 2015 IEEE Energy Conversion Congress and Exposition (ECCE).

[25] Schwack F., Bader N., Leckner J., Demaille C., Poll G. (2020). A study of grease lubricants under wind turbine pitch bearing conditions. Wear.

[26] Srinidhi S., Tiwari M., Burra R., Gowda H., Siemers P.A. Bearing wear due to mechanical stresses and electrical currents. Proceedings of the International Joint Tribology Conference.

[27] Spikes H.A. (2020). Triboelectrochemistry: Influence of applied electrical potentials on friction and wear of lubricated contacts. Tribol. Lett..

[28] Farfan-Cabrera L.I., Erdemir A., Cao-Romero-Gallegos J.A., Alam I., Lee S. (2023). Electrification effects on dry and lubricated sliding wear of bearing steel interfaces. Wear.

[29] Arakere N.K. (2016). Gigacycle rolling contact fatigue of bearing steels: A review. Int. J. Fatigue.

[30] Morales-Espejel G., Gabelli A. (2020). A model for rolling bearing life with surface and subsurface survival: Surface thermal effects. Wear.

[31] Goett G., Gericke A., Henkel K.-M.K.-M., Uhrlandt D. (2020). Determining the arc temperature in submerged arc welding using the Bartels method. J. Phys. D Appl. Phys..

[32] Mashloosh K., Eyre T. (1985). Abrasive wear and its application to digger teeth. Tribol. Int..

[33] Muzyka M.R., Boiko A.V. (2020). Method of Evaluating Residual Stresses in the Product Material. Strength Mater..

[34] Muetze A., Binder A. (2007). Calculation of Motor Capacitances for Prediction of the Voltage Across the Bearings in Machines of Inverter-Based Drive Systems. IEEE Trans. Ind. Appl..

[35] Esmaeili K., Wang L., Harvey T.J., White N.M., Holweger W. (2022). Electrical Discharges in Oil-Lubricated Rolling Contacts and Their Detection Using Electrostatic Sensing Technique. Sensors.

