# Supplementary material for: Formation of Corrugated Damage on Bearing Race under Different AC Shaft Voltages
Source: Materials (Basel). 2024 Feb 12;17(4):859. doi: 10.3390/ma17040859 (PMC10889975; doi:10.3390/ma17040859)
Supplement: Supplementary file 1 [file materials-17-00859-s001.zip › materials-2770018-supplementary.pdf]

Formation of corrugated damage on bearing race under different AC shaft voltages

Lou Zhihao, Song Chenfei*, Ren Yulong, Pang Xianjuan, Lu Huanhuan, Du Sanming, Zhang Yongzhen

National United Engineering Laboratory for Advanced Bearing Tribology, Henan University of Science and Technology, Luoyang 471023, Henan Province, PR China

*E-mail addresses: cfsong@haust.edu.cn

In Figure 5g in the main text, a scratch clearly marks the corrugated damage area. By using a 3D profiler, the scratch mark can also be clearly imaged. By comparing Figure 5 and Figure S1, it can be clearly observed that the high area in the 3D morphology is the light area in the image obtained using optical microscope.

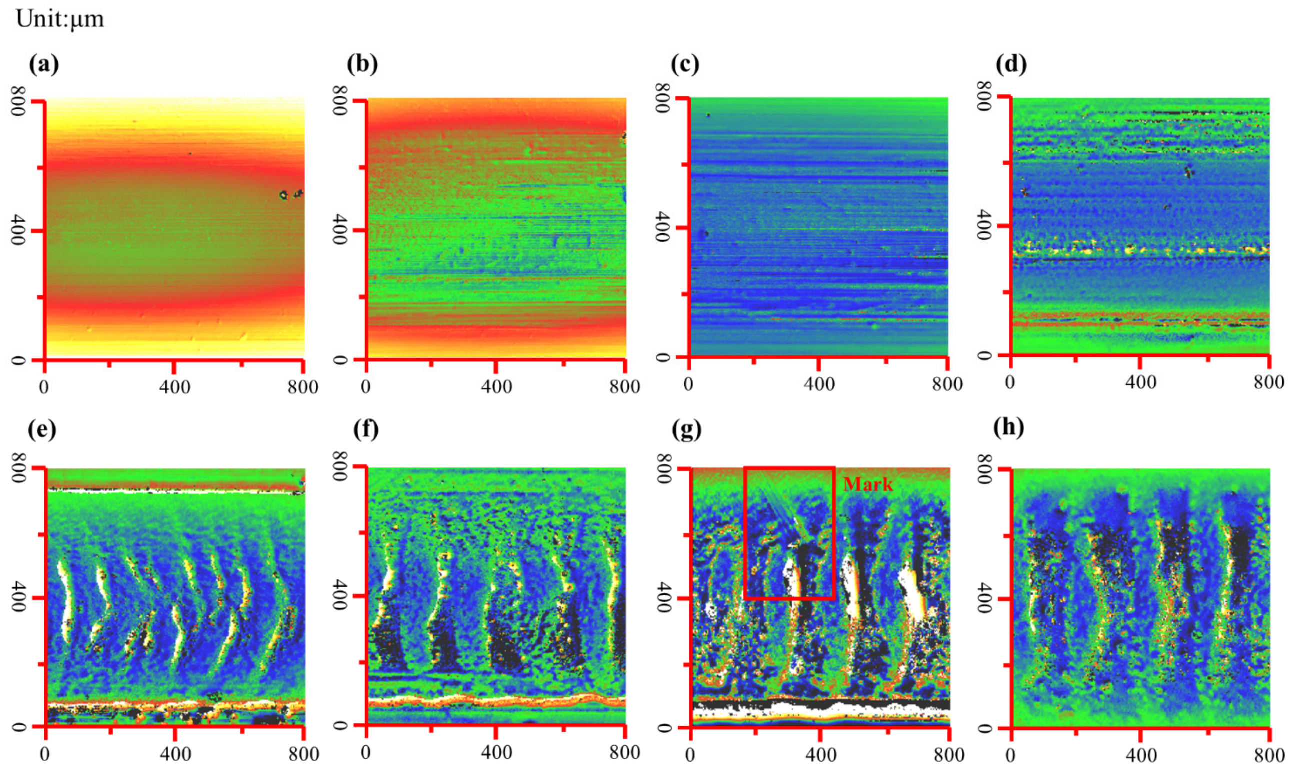

Figure S1. 3D morphology of outer race surface.
(a) Original; (b) 0 V; (c) 1.2 V; (d) 2.2 V; (e) 2.7 V; (f) 3.2 V; (g) 3.7 V; (h) 4.2 V.
